# Normative Data for Macular Thickness and Volume for Optical Coherence Tomography in a Diabetic Population without Maculopathies

**DOI:** 10.3390/jcm12165232

**Published:** 2023-08-11

**Authors:** Carolina Arruabarrena, Antonio Rodríguez-Miguel, Fernando de Aragón-Gómez, Purificación Escámez, Ingrid Rosado, Miguel A. Teus

**Affiliations:** 1 Retina Unit, Department of Ophthalmology, University Hospital “Príncipe de Asturias”, 28805 Alcalá de Henares, Madrid, Spain; 2 Department of Biomedical Sciences, University of Alcalá (IRYCIS), 28805 Alcalá de Henares, Madrid, Spain; 3 Department of Medical Sciences (Ophthalmology), University of Alcalá, 28805 Alcalá de Henares, Madrid, Spain

**Keywords:** diabetic macular edema, diabetic retinopathy, normative macular thickness, optical coherence tomography, screening

## Abstract

Purpose: The purpose was to establish normative data for the macular thicknesses and volume using spectral-domain optical coherence tomography (SD-OCT) in a diabetic population without maculopathies for use as a reference in diabetic retinopathy (DR) and diabetic macular edema screening programs. Methods: This was an observational study nested in a cohort of diabetics from a telemedicine DR screening program. Each patient underwent SD-OCT centered on the fovea. Macular thickness and volume were described and compared using the built-in normative database of the device. Quantile regression models for the 97.5% percentile were fitted to evaluate the predictors of macular thickness and volume. Results: A total of 3410 eyes (mean age, 62.25 (SD, 0.22) years) were included. Mean (SD) central subfield thickness (CST) was 238.2 (23.7) µm, while center thickness (CT), average thickness (AT), and macular volume (MV) were 205.4 (31.6) µm, 263.9 (14.3) µm, and 7.46 (0.40) mm^3^, respectively. Para- and perifoveal thicknesses were clinically and statistically significantly thinner in our population than in the normative reference database. The 97.5% percentile of the thickness of all sectors was increased in males and in the para- and perifovea among those with DR. Conclusions: All ETDRS sectors were thinner in patients with diabetes than in the reference population, except for the CST, which was the most stable parameter that only changed with sex. The upper cutoff limit to detect diabetic macular edema (DME) was different from that of the reference population and was influenced by conditions related to diabetes, such as DR. Therefore, specific normative data for diabetic patients should be used for the screening and diagnosis of DME using SD-OCT.

## 1. Introduction

**Figure 1:**
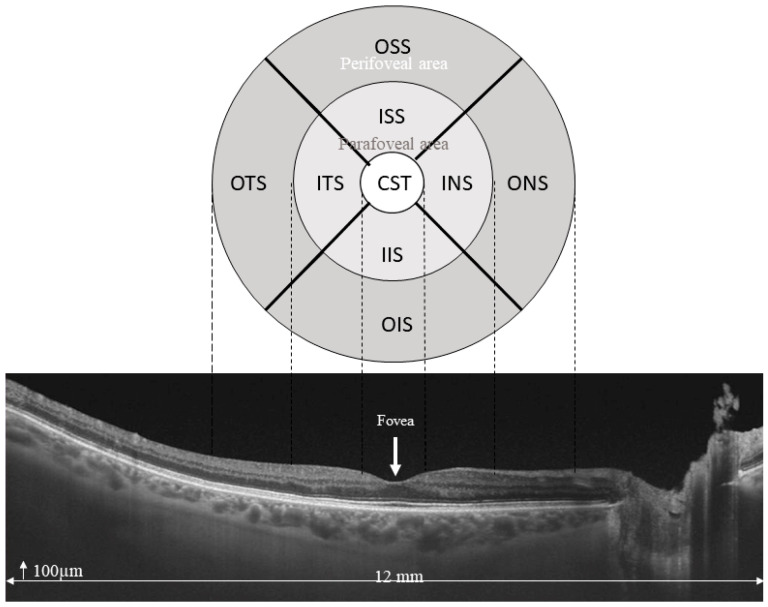
ETDRS map showing CT, CST, inner sectors or parafoveal area (inner temporal sector (ITS), inner nasal sector (INS), inner superior sector (ISS) and inner inferior sector (IIS)), and outer sectors or perifoveal area (outer temporal sector (OTS), outer nasal sector (ONS), outer superior sector (OSS), and outer inferior sector (OIS)).

Optical coherence tomography (OCT) is a non-invasive technique that obtains images of the macula with histological resolution [1,2]. Nowadays, it has become the gold standard for the diagnosis and follow-up of maculopathies because of its high sensitivity, good reliability, and reproducibility [3]. The first temporal domain devices (TD-OCT) have been replaced by spectral domain OCT (SD-OCT) devices that have higher scanning speed, fewer artifacts [4], better axial resolutions, and an acceptable cost. Almost all OCT devices provide similar retinal thickness parameters based on the Early Treatment of Diabetic Retinopathy Study (ETDRS) maps [5]. The central subfield (CST) is the most widely used inclusion and retreatment criteria in clinical trials and practice, including diabetic macular edema (DME) [6], and the other are four inner and outer sectors of the ETDRS map (para and perifovea areas, Figure 1), center thickness (CT), average thickness (AT), and macular volume (MV) [5].

Currently, the thicknesses are calculated using an automatic segmentation software that sets the inner and outer limits and calculates the AT in each of the nine sectors of the ETDRS map. All SD-OCT devices use the internal limiting membrane as the internal limit, but each device uses a different external limit to obtain different thicknesses for the same B-scan [4,7]. Therefore, due to this and other factors such as the refractive index mismatch, resolution, scan numbers used for 2D or 3D scans, dispersion correction methods used to match the system performance, laser power, bandwidth, and central wavelength used for different OCT systems, the macular thickness varies depending on the OCT device employed and the scanning protocol used [7,8,9].

The thicknesses have been measured in a healthy population with different OCT instruments to create a normative database for each instrument. They were compared with each other and with initial TD-OCT in healthy populations [5,7,9] and patients with macular diseases [4,8] to measure reproducibility and create conversion tables [9,10,11].

Macular thickness in healthy people varies according to age [5,12,13,14,15,16], sex [5,13,14,15,16], refractive error [1,2,3,4,5,6,7,8,9,10,11,12,13,14,15,16,17,18,19], and race [16], but it is not clear if in patients with DM, it also changes with the severity of diabetic retinopathy (DR) and the duration of DM [5]. Some studies found no difference between DM patients without retinopathy and healthy subjects, although both studies used TD-OCT and a SD-OCT prototype, with limited resolution [18,19]. However, a decrease in the macular thickness in diabetic patients even without DR, due to diabetic neurodegenerative retinopathy, has recently been described using modern OCT technology [20,21,22,23,24]. Based on the observations from OCT scans of diabetic patients without DR, it seems that some kind of neurodegeneration occurs before DR. Specifically, a reduction in inner retinal layers (ganglion cell layer and nerve fiber layer) in the macular and peripapillary regions has been clearly detected [20,21,22,23,24,25]. In addition, it has been hypothesized that these changes in OCT may be an early marker of systemic ischemic damage [26]. However, there is controversy regarding the influence of DM on total retinal thickness in both old [27] and modern studies [22]. This is due, at least in part, because of the heterogeneity of the study designs, which include type 1 and type 2 diabetics, patients with and without DR, and the different OCT devices used [26]. In addition, there are differences in the duration of DM within subgroups. Nevertheless, it seems that patients with diabetes for more than 10 years may experience an increase in global retinal thickness despite a decrease in the inner retina [22,28].

The Topcon 3SD-OCT Maestro 1 (Topcon Medical Systems, Inc., Oakland, NJ, USA) is a suitable device for screening DR, as it combines a non-mydriatic camera and SD-OCT. Introduced in 2013, it has an analysis software (OCT Data Collector) that obtains the AT in each sector of the ETDRS map, in addition to the MV and overall AT. It uses a reference normative database of macular thicknesses from healthy subjects (published elsewhere) [12,17] in the internal literature. However, at present, there is a lack of specific data on the thicknesses in the diabetic population [12,17].

Specific normality data for individuals with DM are crucial since they establish the threshold for diagnosing DME. If the threshold differs from that of the healthy population, it may lead to misdiagnosis among these patients. Our group conducted a previous study [29] to identify the best DME diagnostic criteria in SD-OCT within a DR screening program. Our research revealed that using as a cutoff either an MV >8 mm^3^ or a thickness of the parafoveal area beyond two standard deviations (SD) of the mean normal value resulted in numerous false positives and a low positive predictive value.

Therefore, we aimed to create a normative database for diabetic patients with different degrees of retinopathy (excluding proliferative diabetic retinopathy (PDR)), without maculopathies or neuropathies, and to evaluate whether other characteristics such as age, sex, DM duration, and degree of retinopathy could act as predictors. Finally, we compared our data with the built-in normative database of the Topcon 3SD-OCT Maestro.

## 2. Materials and Methods

### 2.1. Design and Subject Selection

This observational study was nested in a cohort of diabetics from a telemedicine DR screening program. A randomly sampled eye from one visit of patients with DM referred for ophthalmological screening between 2016 to 2019 was included.

Eligible participants were 18 years old or older with type 1 or type 2 DM sent by their referring doctor for community DR screening. Exclusion criteria were as follows: (1) retinal thickening or thinning due to any macular disease based on fundus photography examination or OCT, (2) macular laser photocoagulation, proliferative DR, or pan-photocoagulation based on grading of fundus photographs, (3) prior treatment for macular edema, (4) glaucoma or other neuropathy based on fundus photography or OCT, (5) OCT scans with artifacts, and (6) OCT scans with a signal strength of less than Top Q 40 [30].

### 2.2. OCT Measurements Procedures and Main Outcomes

**Figure 2:**
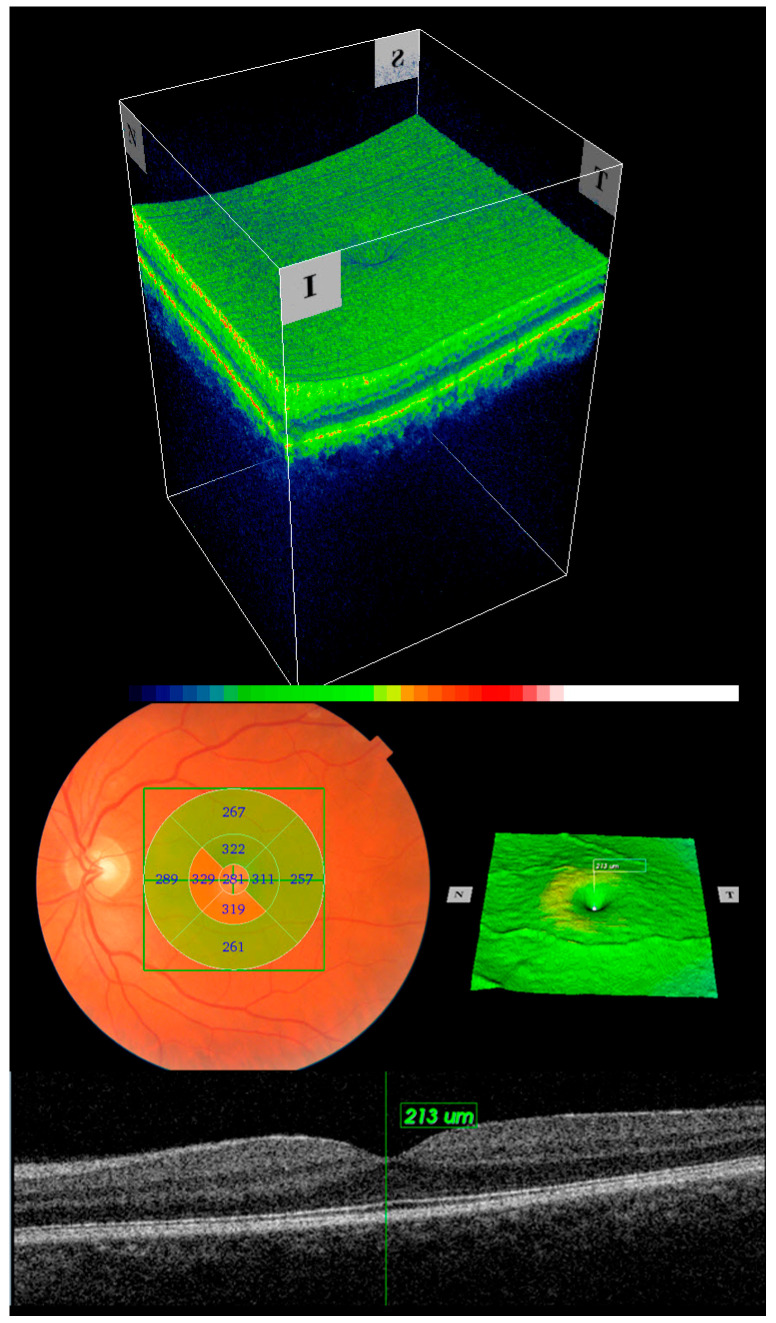
The figure shows a composite image of the 6 × 6 mm cube in 3D, with a 2D B-scan centered in the fovea, a superficial topographic macular map, and fundus photography with the ETDRS map superimposed.

An experienced optometrist imaged all patients with a Topcon 3D SD-OCT Maestro 1, with a lateral and axial resolution of 20 and 6 µm, respectively, using a 6 × 6 mm^2^ 3D macular cube protocol (Figure 2).

For consistent clinical practice, OCT measurements were performed using the default axial length (24.46 mm) and refractive error (0.0 diopters). After acquisition, all macular images were manually checked to ensure that they were free of artifacts (boundary errors and off-centering), and complete cross-sectional images were obtained for all individual line scans. All OCT measurements were performed under non-mydriatic conditions. In cases of poor image quality, the patients were dilated with tropicamide (10 mg/mL) and reimaged.

The instrument software automatically determined the retinal thickness of the macula as the distance between the internal limiting membrane and the signal from the anterior boundary of the retinal pigment epithelium–choriocapillaris region.

The main outcomes (CT, CST, AT, inner and outer ETDRS map sector thicknesses, and MV) were automatically measured on the 6 mm macular thickness map analysis (6 × 6 3D macular cube) and displayed through the OCT data collector software.

### 2.3. Statistical Analysis

Quantitative variables are expressed as mean (±SD) or median and interquartile range (IQR) for non-normally distributed data as well as the range. Qualitative variables are expressed as frequencies and percentages. Differences in quantitative variables between two groups were compared using the Student’s *t*-test, and one-way ANOVA or Kruskal–Wallis tests (for parametric or non-parametric evaluation, respectively) were used for comparisons of three or more groups. Differences in frequencies were compared using the chi-square test or Fisher’s exact test when the assumptions needed for the former were not met. Absolute standardized differences for quantitative and qualitative variables were calculated as measures of imbalance between populations, and a value > 0.40 was considered a high imbalance.

Stratified analyses were performed according to age, sex, type of DM, presence of DR, and time since DM diagnosis.

Quantile regression models were fitted for the 97.5% percentile to evaluate the predictors of macular thickness and volume, and fully adjusted coefficients with their 95% confidence intervals (95%CI) were obtained.

Statistical significance was set at *p* < 0.05 but was adjusted for multiple comparisons when necessary. All analyses were performed using STATA/MP v.17 (Stata Corp LLC., College Station, TX, USA, 2017).

### 2.4. Ethics

The Ethics Committee of the University Hospital Príncipe de Asturias (Alcalá de Henares, Madrid, Spain) approved this study, which was conducted in accordance with the Declaration of Helsinki (Fortaleza 2013).

## 3. Results

### 3.1. Selection of Eyes, Baseline Clinical Characteristics of the Eyes Included, Overall and by Other Comorbidities

**Figure 3:**
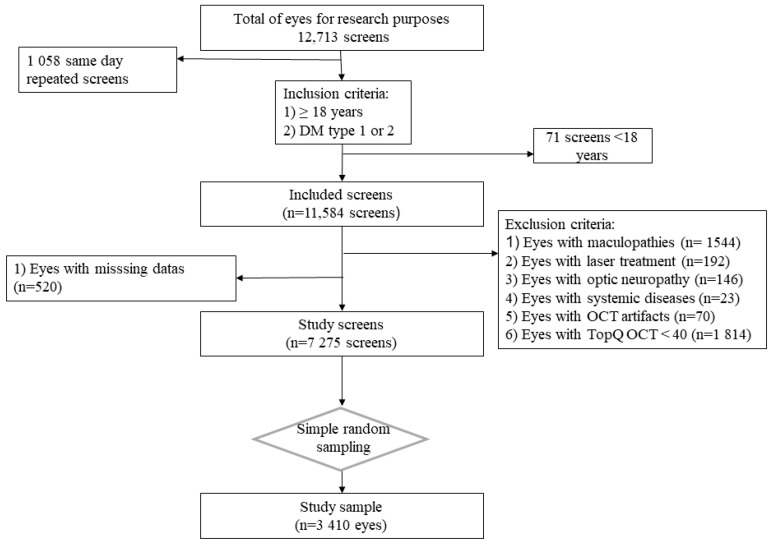
Flowchart of study sample inception.

From 2016 to 2019, 7275 screens met the eligibility criteria (Figure 3), and 1 eye from each patient was randomly selected; thus, finally, 3410 eyes were included in the study.

The patients were 57.7% males, with a mean age of 62.2 ± 12.8 years (range, 18–93 years), and 3078 (90.2%) patients had a BCVA ≥ 6/12. Most patients were diagnosed with type 2 DM with a time from the diagnosis lower than 15 years. Among the patients, 2849 (83.5%) had no DR, and 14 eyes (0.5%) had severe non-proliferative DR. More than 60% (2137) of the patients had hypertension and hypercholesterolemia (2292). A total of 684 (20%) patients were current smokers, 396 (11.6%) had a history of acute myocardial infarction, 20 (0.6%) had a history of stroke, and 391 eyes (11.5%) were reimaged after pharmacological mydriasis (Table 1).

### 3.2. Macular Thickness and Volume Measured with SD-OCT in DM Patients without Maculopathies

Mean (SD) CST, CT, AT, and MV were 238.2 (23.7) µm, 205.4 (31.6) µm, 263.9 (14.3) µm, and 7.46 (0.40) mm^3^, respectively (Table 2). Overall thickness was greater within the inner macula, nasal was the thickest (299.0 µm; SD: 17.4), followed by superior, inferior, and temporal, which were 296.6 (18.4) µm, 292.9 (18.1) µm, and 285.1 (16.8) µm, respectively. Within the outer macula, the order was similar, but mean (SD) thicknesses were thinner: 272.0 (16.1) µm, 255.0 (16.3) µm, 253.2 (16.2) µm, and 244.0 (17.5) µm (Table 2). As Figure 1 displayed, our study showed a distinct topography where the retinal thickness is thinnest in the fovea and thicker in the parafoveal area.

### 3.3. Comparison of the Macular Thickness Measured with SD-OCT in DM Patients without Maculopathies with the Reference Normative Database of Topcon 3SD Maestro

The parafoveal area was thinner in our population than in the reference: ITS, 285.1 (16.8) µm vs. 296.59 (16.62) µm; ISS, 296.6 (18.4) µm vs. 308.98 (16.19) µm; INS, 299.0 (17.4) µm vs. 309.33 (16.68) µm; IIS, 292.9 (18.1) µm vs. 305.73 (16.32) µm; and even thinner in DM without DR. The absolute standardized difference was >0.59 for all sectors, meaning a high imbalance between populations (Table 3). Similar results were observed within the perifovea, with an absolute standardized difference ≥ 0.52 for all sectors except the OIS, which was 0.33 (Table 3). In contrast, CST was statistically significantly thicker in our population than in the reference: the mean (SD) was 238.2 (23.7) µm vs. 234 (20.65) µm, respectively, and even thicker in our population with DR: the mean (SD) was 239.0 (25.4) µm, although the standardized absolute difference was 0.18 (Table 3).

### 3.4. Baseline Characteristics and Comorbidities Associated with Macular Thickness and Volume in DM Patients without Maculopathies

Regarding the 97.5% percentile of CT, male sex, age > 60 years, history of stroke, and dyslipidemia were associated with increased thickness, while image quality and the need for pupil dilation were associated with decreased thickness (Table 4). The 97.5% percentile of the CST was associated with an increased thickness among males and a decrease depending on the quality of the images (Table 4). In contrast, the 97.5% percentile of the para- and perifoveal areas increased in males and among those with DR, while age showed a trend toward decreased thickness, especially among those older than 71 years (Table 4). The 97.5% cutoff value of MV was influenced only by sex and age > 71 years (Table 4).

Similar results were observed when stratifying by type of DM: males were associated with an increased thickness in all sectors and volume for the 97.5% percentile, across both strata, except for CT in type 1 DM, which did not reach statistical significance, while age, especially among those with type 2 DM ≥ 71 years was associated with a decreased 97.5% percentile (Table 5).

**Figure 4:**
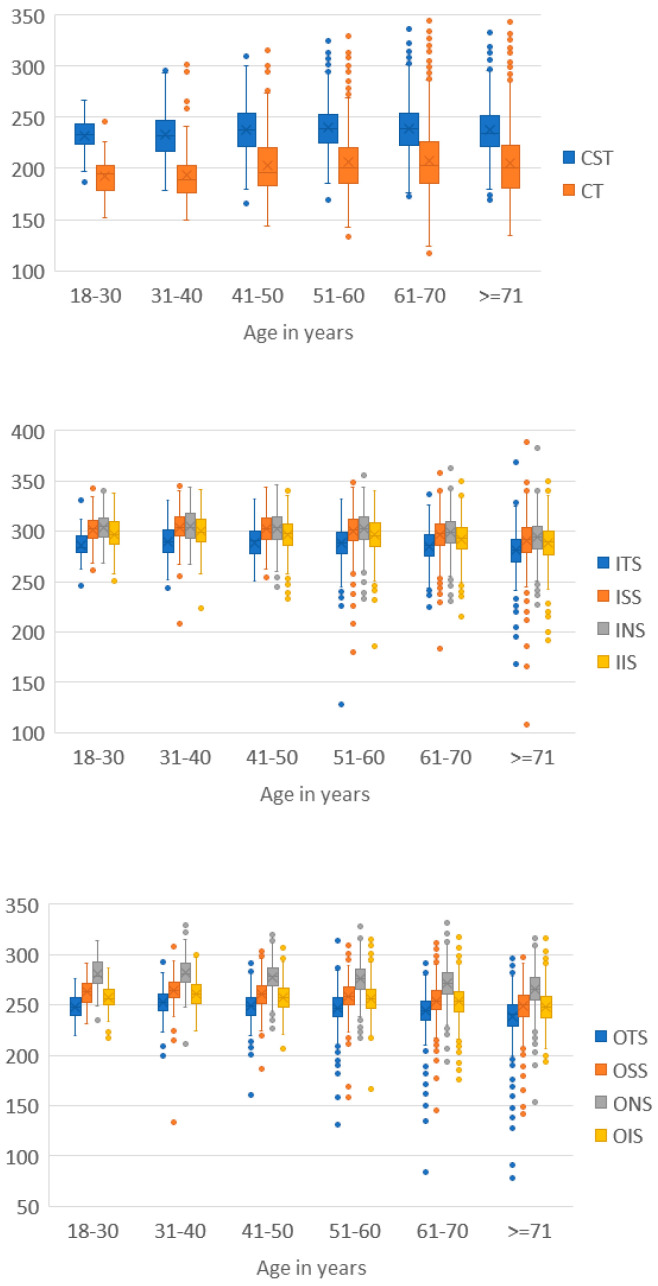
Median ETDRS map sector thickness by groups of age. The horizontal line (_) within the box represents the median, and the cross within the box (X) represents the mean.

The median (IQR) thickness of the ETDRS sectors stratified by age showed a decreasing trend (nasal > superior > inferior > temporal), remained stable until 60 years of age, and then decreased. CST, CT, and MV increased after 40 years, reached a maximum at 51–60 years, and decreased thereafter (Figure 4). Therefore, the comparison between those younger and those older than 60 years was statistically significant for MV and all thicknesses, except for CST (Supplementary Table S1).

We found statistically significant differences according to sex; they were lower in females (Supplementary Table S2). Among eyes with DR, we found a slight but statistically significant increase in MV, perifoveal thickness, and AT compared to those without (Supplementary Table S3).

The main outcomes were statistically significantly thinner among subjects with Type 2 DM than in type 1 and in those with ≥15 years of DM follow-up, except CST and CT, which were related to greater values in type 2 DM Supplementary Tables S4 and S5).

## 4. Discussion

We found that the cutoff values at the upper limit of normal macular thicknesses are not only influenced by some characteristics, such as sex and age, in diabetic patients without maculopathies, as well as in the general population, but also depend on image quality [30] and pupil dilation. Moreover, the cutoff thickness of the para- and perifoveal sectors increases in diabetics who present with DR. These data could explain the high percentage of false positives that we found in a previous study [29] for DR when we used an increase beyond 2SD from the mean of the reference population in the fovea and parafovea as a detection criterion. In the current study, we found that 45.3% of the false positives [29] when using the foveal and parafoveal thickening criteria were in patients with a normal macular profile that exceeded the thickness beyond 2SD from the mean of the reference population. If we use the new normative values for the healthy diabetic population, the number of false positives in the DR screening program would decrease, and the diagnostic profile of this quantitative criterion, which is simple to obtain, easy, and quick to evaluate, would improve. Apart from the CST, healthy patients with DM differ significantly from healthy patients and should use a special normative database for SD-OCT.

The thickness of the CST and CT is the least influenced by DM, not presenting statistically significant differences either by the type of DM, the time since the diagnosis of DM, or the presence of DR. CST only showed statistically significant differences according to sex, as shown in other studies in DM patients without maculopathy [14,15,18]. This means that CST is probably the most reliable parameter for evaluating the changes induced by treatments or interventions, but it must be considered with different limits for each sex; males have significantly thicker parameters on OCT, not only in DM patients [18,22] but also in healthy patients [12,13,14,15,17,19]. Invernizzi et al. [16] attributed this difference to the thicker inner and outer nuclear layers present in males.

Topographically, there is a pattern of thickness distribution: the foveal center is the thinnest in patients with DM without maculopathy, and the parafoveal area is the thickest. In our study, we found that the nasal sector was the thickest of the para- and perifoveal regions, followed by the superior, inferior, and temporal sectors (Table 2). These patterns were maintained in both sexes and all age groups (Supplementary Material Tables S1 and S2). Previous studies [14,15] using different types of OCT or retinal thickness analyzers in healthy populations and patients with DM (without maculopathy) [14] also reported a similar pattern of macular thickness that might be related to the crowding of nerve fibers in the parafoveal region and along the papillomacular bundle in the perifovea [15,16].

The effect of aging on macular thickness measured through OCT has revealed controversial results [13,15,19] that motivated a systematic review of the literature [31]. In our study, a characteristic pattern of changes in OCT thickness with age has been demonstrated in the DM population. The thicknesses of the peri- and parafovea remained stable until 51–60 years and decreased progressively thereafter. While the CST and CT remained stable between the ages of 18 and 40 years, they increased progressively until the age range of 61 to 70 years and then began to decrease (Figure 3). Similar data have been reported in healthy populations [13,14,15,16,31]. It is believed that cells in the foveal area (cones and RPE cells) remain stable until old age, when metabolic and phagocytic processes increase and RPE cells become thicker. However, the para- and perifoveal regions are composed of more cell layers, especially ganglion cells and nerve fiber layers, which diminish with age.

Although initial studies in patients with DM without DR did not show differences from healthy subjects [18,19], other studies have revealed differences in the OCT thicknesses of patients with DM (without maculopathy) [22,28]. This is probably due to the lower precision of the first OCT instrument (TD-OCT), which did not allow small differences to be detected. Our normative database of patients with DM without maculopathies, as in other previous studies [22,23,24,25], showed leaner parameters than the Topcon 3SD OCT1-Maestro normative database for healthy subjects [12,17] (Table 3). An increase in the CST in the group of healthy DM patients compared to the Topcon normative base was statistically significant (*p* < 0.025), both for DM patients without DR and, more importantly, for healthy DM patients with DR. Similar data have been found in other study [22] that describes an increase in the total macular thickness, especially at the central area in DM patients with DR, and a decrease in the thickness of the internal retina, which would mainly affect the para- and perifoveal area. We also found a decrease in the thickness in all sectors of the peri- and parafovea in patients with DM without maculopathies that were both clinically relevant (standardized absolute differences greater than 0.5) and statistically significant (*p* < 0.025) in DM eyes with and without DR. Neuronal degeneration is a likely explanation for the differential macular thinning in patients with diabetes because the most affected layer in this neurodegeneration is the nerve fiber layer [20,21,22,23,24,25]. This layer is almost absent in the central macula and thicker in the parafovea, thus making this area more sensitive to reflect changes in the nerve fiber thickness. There is a study that suggests that neurodegeneration and a decrease in nerve fiber layer thickness appear early in the disease. Later, the retina may show an increase in the total retinal thickness over time due to the vascular injury and the increased vascular permeability and edema that the DM may cause in the retina [22].

Other factors to consider are that the different types of DM [23,24] and duration of the disease [22,28] present differences in the distribution of some characteristics, which could bias the results, and that there may be thinning of the macula due to ischemia that could interfere with the results [24] that we cannot analyze in this study since we have not performed Fluorescein angiography or Angio-OCT to evaluate it.

When we analyzed the different characteristics related to DM that can influence thickness, we only found clinically relevant and statistically significant differences in general parameters between type 1 and type 2 DM (Supplementary Table S4). Differences between patients with and without DR (Supplementary Table S3), or with more or less than 15 years of DM evolution (Supplementary Table S5), although statistically significant, have little clinical relevance.

The strength of the current study is that it is the first to provide normative macular thickness data using the Topcon 3SD-OCT1 Maestro device in a large sample of patients with DM without maculopathy. Furthermore, the wide age range of our sample provides a good representation of older and younger adults in clinical practice. Therefore, our data provide a benchmark for clinicians to assess and compare macular changes in patients with DM, particularly those with DME. The normative database with which the Topcon 3D- OCT Maestro was marketed was based on a sample of 115 healthy patients. In 2018, a new normative database for the instrument was established using a sample of 399 subjects studied in 2015 [13]. This sample included only 35 subjects over 70 years of age and a sparse representation of patients with DM, although the macular thicknesses used by the instrument to set the cutoff limits for DME detection were based on these data.

Our study has some limitations: we used the default axial length (24.46 mm) and refraction (0.0 diopters) to capture the scans, although this should not affect the thickness values apart from a slight overestimation in the perifovea. Refractive errors and axial length were not measured, although eyes with high myopia, which seem to induce most artifacts [30], were excluded. Finally, our patients were Caucasians, and it would be interesting to include patients of other ethnicities.

## Figures and Tables

**Table 1:** Clinical and sociodemographic characteristics of the population.

	Overall (*n* = 3410)
**Age.** mean (SD)	62.25 (0.22)
**Gender** male *n*, (%)	1968 (57.7%)
**Right eye** *n*, (%)	1668 (48.9%)
**CV risk factors**, *n* (%):	
Current smoker	684 (20.0%)
Hypertension	2137 (62.7%)
Dyslipidemia	2292 (67.2%)
History of acute myocardial infarction	396 (11.6%)
History of stroke	20 (0.59%)
**VA categorized by groups**, *n*, (%)	
≤0.3	1237 (36.3%)
0.4–0.5	253 (7.42%)
≥0.5	1920 (56.3%)
**Duration of diabetes**, in years, *n* (%):	
≤15	2552 (74.8%)
>15	858 (25.2%)
**DM type 2**, *n* (%)	3135 (91.9%)
**Diabetic retinopathy**, * *n* (%):	
No abnormalities	2849 (83.5%)
Mild non-proliferative	362 (10.6%)
Moderate non-proliferative	185 (5.42%)
Severe non-proliferative	14 (0.47%)
**Sight-threatening DR**, *n* (%):	
No abnormalities	2849 (83.5%)
ST-DR	14 (0.47%)
No ST-DR	547 (16.0%)
**Dilated**, *n* (%)	391 (11.5%)

DM: diabetes mellitus, SD. Standard deviation, CV: Cardiovascular, VA: Visual acuity, DR: diabetic retinopathy, ST-DR: Sight-threatening diabetic retinopathy. * According to ICO grading system.

**Table 2:** Normative Data for ETDRS Macular Thickness (in µm) and Volume (in mm^3^) measured by Spectral-Domain Optical Coherence Tomography (Topcon 3SD OCT Master).

*n* = 3410	Center Macula		Inner Macula				Outer Macula				Average Thickness	Total Volume
	CST	Center	Temporal	Superior	Nasal	Inferior	Temporal	Superior	Nasal	Inferior		
Median	237.3	200	285.6	297.6	299.3	293.4	244.8	255.5	272.4	253.0	264.3	7.47
IQR	222.6–252.7	183–222	274.6–296.3	286.0–308.2	287.9–310.6	282.0–304.7	235.1–254.4	245.1–265.2	261.4–282.9	243.2–263.5	254.9–273.2	7.21–7.72
Mean	238.2	205.4	285.1	296.6	299.0	292.9	244.0	255.0	272.0	253.2	263.9	7.46
SD	23.7	31.6	16.8	18.4	17.4	18.1	17.5	16.3	16.1	16.2	14.3	0.40
Min–Max	165.8–336.1	117–345	128.5–368.0	107.7–388.6	227.2–382.3	185.9–350.0	178.5–314.0	133.2–310.9	153.2–331.4	166.0–317.0	190.9–313.4	5.40–8.86
95%CI	237.4–239.0	204.3–206.4	284.5–285.7	295.9–297.2	298.5–299.6	292.3–293.5	243.4–244.6	254.4–255.5	271.4–272.6	252.7–253.8	263.4–264.3	7.44–7.47

ETDRS: Early treatment Diabetic retinopathy study; CST: Central subfield thickness; SD: standard deviation; IQR: Interquartile range; CI: confidence interval; min: minimum; and max: maximum.

**Table 3:** Comparison between Topcon normative database of healthy people and our normative database of diabetic people without maculopathies.

		Mean (SD), µm					
		Arruabarrena et al. *n* = 3410	Arruabarrena et al. no DR *n* = 2849	Arruabarrena et al. DR *n* = 561	Chaglasian et al. [12] + Normative Topcon 3D SD-OCT Maestro N = 395	Absolute Standardized Difference	*p* Value
Center macula	CST	238.2 (23.7)	238.0 (23.4)	239.0 (25.4)	234 (20.65)	0.18 ^1^ 0.17 ^2^ 0.21 ^3^	**<0.001 ^1^** **<0.001 ^2^** **<0.001 ^3^**
Inner macula	Temporal	285.1 (16.8)	284.9 (16.6)	286.0 (17.7)	296.59 (16.62)	**0.68 ^1^** **0.70 ^2^** **0.61 ^3^**	**<0.001 ^1^** **<0.001 ^2^** **<0.001 ^3^**
	Superior	296.6 (18.4)	296.5 (18.2)	296.8 (19.5)	308.98 (16.19)	**0.68 ^1^** **0.69 ^2^** **0.67 ^3^**	**<0.001 ^1^** **<0.001 ^2^** **<0.001 ^3^**
	Nasal	299.0 (17.4)	299.0 (17.2)	298.8 (18.3)	309.33 (16.68)	**0.59 ^1^** **0.60 ^2^** **0.60 ^3^**	**<0.001 ^1^** **<0.001 ^2^** **<0.001 ^3^**
	Inferior	292.9 (18.1)	292.8 (17.8)	293.5 (19.5)	305.73 (16.32)	**0.72 ^1^** **0.74 ^2^** **0.67 ^3^**	**<0.001 ^1^** **<0.001 ^2^** **<0.001 ^3^**
Outer macula	Temporal	244.0 (17.5)	243.6 (17.2)	246.1 (18.5)	252.93 (13.94)	**0.52 ^1^** **0.55 ^2^** **0.41 ^3^**	**<0.001 ^1^** **<0.001 ^2^** **<0.001 ^3^**
	Superior	255.0 (16.3)	254.5 (16.0)	257.2 (17.4)	269.50 (15.16)	**0.90 ^1^** **0.94 ^2^** **0.74 ^3^**	**<0.001 ^1^** **<0.001 ^2^** **<0.001 ^3^**
	Nasal	272.0 (16.1)	271.8 (16.5)	273.1 (18.1)	284.15 (16.42)	**0.75 ^1^** **0.75 ^2^** **0.63 ^3^**	**<0.001 ^1^** **<0.001 ^2^** **<0.001 ^3^**
	Inferior	253.2 (16.2)	252.9 (16.0)	254.6 (17.2)	258.58 (14.90)	0.33 ^1^ 0.35 ^2^ 0.24 ^3^	**<0.001 ^1^** **<0.001 ^2^** **<0.001 ^3^**

^1^ Arruabarrena vs. Chaglasian; ^2^ Arruabarrena No DR vs. Chaglasian (statistically significant *p*-value = 0.025); ^3^ Arruabarrena DR vs. Chaglasian (statistically significant *p*-value = 0.025). SD: standard deviation; DR: diabetic retinopathy; SD-OCT: spectral-domain optical coherence tomography; CST: central subfield thickness; CI: confidence interval.

**Table 4:** Factors associated with macular thickness and volume in 97.5% percentile, by quantile regression.

CT, CST, and Volume					
	Fully Adjusted Coefficients (95%CI)				
97.5% Percentile	CT µm		CST µm		MV mm^3^
**Gender:**					
Females	Ref.		Ref.		Ref.
Males	**9.80 (2.35, 17.2)**		**10.7 (4.51, 17.0)**		**0.13 (0.06, 0.20)**
**Age**, years:					
≤60	Ref.		Ref.		Ref.
61–70	**13.4 (4.43, 22.4)**		2.16 (−5.34, 9.66)		0.09 (−0.18, 0.003)
≥71	**15.6 (5.68, 25.5)**		7.38 (−0.92, 15.7)		**−0.17 (−0.27, −0.07)**
**Laterality:**					
Left eye	Ref.		Not a predictor		Not a predictor
Right eye	−7.00 (−14.3, 0.33)				
**Time since DM diagnosis, in years:**					
≤5	Not a predictor		Not a predictor		Ref.
6–10					−0.03 (−0.13, 0.07)
≥10					−0.05 (−0.14, 0.04)
**TopQ**, quartiles	**−5.40 (−8.86, −1.94)**		**−4.97 (−7.85, −2.08)**		Not a predictor
**Visual acuity**, decimal Snellen:					Not a predictor
0.02–0.4	Not a predictor		Not a predictor		
0.5–1					
**Pupil dilation:**					Not a predictor
No	Ref.		Ref.		
Yes	**−12.4 (−24.1, −0.68)**		−7.31 (−17.1, 2.50)		
**DR:**					
No abnormalities	Not a predictor		Not a predictor		Ref.
ST and non-ST					0.09 (−0.01, 0.19)
**Antecedents of:** *					
Hypertension	Not a predictor		Not a predictor		−0.07 (−0.14, 0.01)
Acute myocardial infarction	Not a predictor		Not a predictor		Not a predictor
Stroke	**54.2 (6.29, 102.1)**		39.9 (−0.16, 80.0)		Not a predictor
Dyslipidemia	**8.20 (0.18, 16.2)**		5.25 (−1.46, 12.0)		Not a predictor
Current exmoker	Not a predictor		Not a predictor		Not a predictor
**Average parafoveal and perifoveal area**					
		**Fully Adjusted Coefficients (95%CI), µm**			
**97.5% Percentile**		**Parafoveal Area**		**Perifoveal Area**	
**Gender:**					
Females		Ref.		Ref.	
Males		**5.80 (3.19, 8.42)**		**5.11 (2.84, 7.37)**	
**Age**, years:					
≤60		Ref.		Ref.	
61–70		−1.08 (−4.18, 2.02)		**−4.68 (−7.36, −2.00)**	
≥71		−4.65 (−7.98, −1.32)		**−8.02 (−10.9, −5.13)**	
**Laterality:**					
Left eye		Not a predictor		Ref.	
Right eye				**−3.13 (−5.34, −0.92)**	
**Time since DM diagnosis**, in years:					
≤5		Not a predictor		Not a predictor	
6–10					
≥10					
**TopQ**, quartiles		Not a predictor		Not a predictor	
**Visual acuity**, decimal Snellen:					
0.02–0.4		Not a predictor		Not a predictor	
0.5–1					
**Pupil dilation:**					
No		Not a predictor		Not a predictor	
Yes					
**DR:**					
No abnormalities		Ref.		Ref.	
ST and non-ST		**5.50 (2.03, 8.96)**		**3.85 (0.87, 6.83)**	
**Antecedents of:** *					
Hypertension		**−4.72 (−7.51, −1.94)**		−1.59 (−4.00, 0.83)	
Acute myocardial infarction		Not a predictor		−2.70 (−6.24, 0.85)	
Stroke		Not a predictor		Not a predictor	
Dyslipidemia		Not a predictor		Not a predictor	
Current smoker		Not a predictor		Not a predictor	

CI: confidence interval; µM: microns; DM: diabetes mellitus; TopQ: quality of the scan; DR: diabetic retinopathy; ST: sight-threatening; and Ref.: reference. * The category of reference is no presence of the disease.

**Table 5:** Factors associated with 97.5% cutoff level of macular central thickness and volume, by DM type. Factors associated with 97.5% cutoff level of parafoveal and perifoveal thickness, by DM type.

	97.5% Cutoff Level, Fully Adjusted Coefficient (95%CI)								
	CT, µm			CST, µm			Macular Volume, mm^3^		
	DM Type 1	DM Type 2		DM Type 1		DM Type 2	DM Type 1		DM Type 2
**Gender:**									
Males	22.00 (−12.23, 56.23)	**9.80 (1.13, 18.47)**		**20.21 (3.56, 36.85)**		**11.10 (4.55, 17.65)**	**0.36 (0.23, 0.49)**		**0.12 (0.05, 0.19)**
**Age**, years:									
61–70	−9.00 (−97.93, 79.93)	**12.00 (1.60, 22.40)**		22.59 (−21.26, 66.44)		2.16 (−5.72, 10.04)	−0.20 (−0.54, 0.13)		−0.06 (−0.14, 0.02)
≥71	53.00 (−90.05, 196.05)	**11.80 (0.33, 23.27)**		35.91 (−33.39, 105.21)		7.02 (−1.63, 15.67)	**−1.04 (−1.58, −0.51)**		**−0.14 (−0.23, −0.05)**
**Time since DM diagnosis**, in years:									
6–10	Not a predictor			Not a predictor			0.13 (−0.11, 0.38)		−0.02 (−0.11, 0.07)
≥10							0.17 (−0.02, 0.36)		**−0.05 (−0.13, 0.03)**
**TopQ**, quartiles	1.00 (−16.56, 18.56)	**−5.40 (−9.39, −1.41)**		−4.16 (−12.81, 4.49)		**−4.85 (−7.86, −1.83)**	Not a predictor		
**Visual acuity**, Snellen decimal:									
0.5–1	−4.00 (−131.86, 123.86)	−13.00 (−32.15, 6.15)		Not a predictor			Not a predictor		
**Pupil dilation:**							Not a predictor		
Yes	−3.00 (−118.01, 112.01)	**−14.00 (−27.14, −0.85)**		6.49 (−50.19, 63.17)		−8.21 (−18.17, 1.76)			
DR:									
ST and non-ST	Not a predictor			Not a predictor			0.08 (−0.05, 0.22)		0.07 (−0.03, 0.17)
**Antecedents of:**									
Hypertension	Not a predictor	Not a predictor		Not a predictor		Not a predictor	0.17 (−0.02, 0.36)		−0.06 (−0.13, 0.01)
Acute myocardial infarction	Not a predictor	Not a predictor		Not a predictor		Not a predictor	Not a predictor		Not a predictor
Stroke	Not a predictor	**54.00 (0.79, 107.21)**		Not a predictor		Not a predictor	Not a predictor		Not a predictor
Dyslipidemia	−3.00 (−42.21, 36.21)	8.20 (−1.23, 17.63)		4.11 (−15.21, 23.43)		39.57(−0.78, 79.92)	Not a predictor		Not a predictor
Current smoker	Not a predictor	Not a predictor		Not a predictor		6.01 (−1.15, 13.16)	−0.02 (−0.16, 0.11)		0.04 (−0.05, 0.13)
	**97.5% Cutoff Level, Fully Adjusted Coefficients (95%CI), µm**								
	**Average Parafoveal Area**				**Average Perifoveal Area**				
	**DM Type 1**		**DM Type 2**		**DM Type 1**			**DM Type 2**	
**Gender:**									
Males	**16.53 (11.28, 21.78)**		**5.39 (2.53, 8.24)**		**6.51 (0.13, 12.89)**			**3.69 (1.24, 6.15)**	
**Age, years:**									
61–70	2.95 (−11.17, 17.08)		−0.77 (−4.15, 2.60)		−1.72 (−18.66, 15.23)			**−4.52 (−7.41, −1.64)**	
≥71	−14.16 (−36.66, 8.35)		**−4.40 (−8.00, −0.81)**		−26.00 (−54.87, 2.87)			**−7.32 (−10.41, −4.23)**	
**Time since DM diagnosis**, in years:									
6–10	Not a predictor				Not a predictor				
≥10									
**TopQ**, quartiles	Not a predictor				Not a predictor				
**Visual acuity**, Snellen decimal:									
0.5–1	Not a predictor				Not a predictor				
**Pupil dilation:** Yes	Not a predictor				Not a predictor				
**DR:**									
ST and non-ST	−0.61 (−6.07, 4.84)		**5.86 (1.93, 9.80)**		1.04 (−5.48, 7.57)			1.33(−2.02, 4.69)	
**Antecedents of:**									
Hypertension	6.26 (−1.00, 13.52)		**−4.87 (−7.91, −1.83)**		2.64 (−6.13, 11.42)			−2.10 (−4.71, 0.52)	
Acute myocardial infarction	Not a predictor		Not a predictor		−11.05 (−34.47, 12.36)			−2.06 (−5.75, 1.63)	
Stroke	Not a predictor		Not a predictor		Not a predictor			Not a predictor	
Dyslipidemia	Not a predictor		Not a predictor		Not a predictor			Not a predictor	
Current smoker	Not a predictor		Not a predictor		Not a predictor			Not a predictor	

CI: confidence interval; µM: microns; DM: diabetes mellitus; TopQ: quality of the scan; DR: diabetic retinopathy; ST: sight-threatening. Reference categories: Gender: female; Age: <60 years; Time since DM diagnosis: <5 years; Visual acuity: <0.5; Pupil dilatation: no; DR: No abnormalities; Antecedent of: no presence of the disease.

## Data Availability

The data presented in this study are available in the article or Supplementary Material here.

## Supplementary Materials

The following supporting information can be downloaded at: https://www.mdpi.com/article/10.3390/jcm12165232/s1, Supplementary Table S1. Normative Data for ETDRS Macular Thickness (in µm) and volume (in mm^3^) measured through Spectral-Domain Optical Coherence Tomography (Topcon 3SD OCT Master), by age; Supplementary Table S2. Normative Data for Macular Thickness (in µm) and volume (in mm^3^) measured through Spectral-Domain Optical Coherence Tomography (Topcon 3SD OCT Master), by gender; Supplementary Table S3. Normative Data for Macular Thickness (in µm) and volume (in mm^3^) measured through Spectral-Domain Optical Coherence Tomography (Topcon 3SD OCT Master), by DR existence; Supplementary Table S4. Normative Data for Macular Thickness (in µm) and volume (in mm^3^) measured through Spectral-Domain Optical Coherence Tomography (Topcon 3SD OCT Master), by type of DM; Supplementary Table S5. Normative Data for Macular Thickness (in µm) and volume (in mm^3^) measured through Spectral-Domain Optical Coherence Tomography (Topcon 3SD OCT Master), by the years since DM diagnosis.
