# Supplementary material for: Normative Data for Macular Thickness and Volume for Optical Coherence Tomography in a Diabetic Population without Maculopathies
Source: J Clin Med. 2023 Aug 11;12(16):5232. doi: 10.3390/jcm12165232 (PMC10455588; doi:10.3390/jcm12165232)
Supplement: Supplementary file 1 [file jcm-12-05232-s001.zip › jcm-2507263-supplementary.pdf]

Supplementary Table 1. Normative Data for ETDRS Macular Thickness (in µm) and volume (in mm^3) measured by Spectral-Domain Optical Coherence Tomography (Topcon 3SD OCT Master), by age.

	**≤ 60 years** (N=1333)	**>60 years** (N=2077)	**Absolute standardized difference**	**p-value**
**ETDRS macular thickness and volume,** mean (SD); 95% CI				
CST	237.9 (22.7); 236.7-239.1	238.4 (24.4); 237.3-239.4	0.02	0.59
Center	203.1 (29.0); 201.6-204.7	206.8 (33.1); 205.4-208.2	0.12	**<0.001**
Inner temporal	288.0 (16.6); 287.2-288.9	283.2 (16.7); 282.5-283.9	0.29	**<0.001**
Inner superior	301.2 (17.8); 300.2-302.1	293.6 (18.5); 292.8-294.4	**0.42**	**<0.001**
Inner nasal	302.9 (16.7); 302.0-303.8	296.5 (17.4); 295.7-297.2	0.38	**<0.001**
Inner inferior	296.7 (17.5); 295.7-297.6	290.5 (18.0); 289.7-291.2	0.35	**<0.001**
Outer temporal	248.0 (15.7); 247.1-248.8	241.4 (18.0); 240.6-242.2	0.39	**<0.001**
Outer superior	260.0 (15.4); 259.2-260.8	251.7 (16.0); 251.0-252.4	**0.53**	**<0.001**
Outer nasal	277.2 (15.5); 276.4-278.0	268.6 (16.7); 267.9-269.4	**0.53**	**<0.001**
Outer inferior	256.9 (15.0); 256.1-257.7	250.9 (16.5); 250.1-251.6	0.38	**<0.001**
Average thickness	268.1 (13.5); 267.3-268.8	261.2 (14.2); 260.6-261.8	**0.50**	**<0.001**
Total volume	7.57 (0.38); 7.55-7.59	7.38 (0.40); 7.36-7.40	**0.50**	**<0.001**

ETDRS: Early Treatment Diabetic Retinopathy Study; SD: standard deviation; CST: central subfield thickness; CI: confidence interval.

Supplementary Table 2. Normative Data for Macular Thickness (in µm) and volume (in mm^3) measured by Spectral-Domain Optical Coherence Tomography (Topcon 3SD OCT Master), by gender.

	**Male** (*N*=1967)	**Female** (*N*=1443)	**Absolute standardized difference**	**p-value**
**ETDRS macular thickness and volume,** mean (SD); 95% CI				
CST	243.3 (22.3); 242.3 - 244.3	231.2 (23.8); 230.0 - 232.4	**0.52**	**<0.001**
Center	209.5 (31.4); 208.1 - 210.9	199.7 (30.9); 198.1 - 201.3	0.31	**<0.001**
Inner temporal	288.6 (16.1); 287.9 - 289.3	280.3 (16.5); 279.5 - 281.2	**0.51**	**<0.001**
Inner superior	299.2 (18.3); 298.4 - 300.0	293.0 (18.0); 292.1 - 293.9	0.34	**<0.001**
Inner nasal	302.5 (17.1); 301.7 - 303.2	294.3 (16.7); 293.4 - 295.2	**0.48**	**<0.001**
Inner inferior	296.5 (17.5); 295.7 - 297.3	288.0 (17.7); 287.0 - 288.9	**0.41**	**<0.001**
Outer temporal	247.0 (16.0); 246.3 - 247.7	239.9 (18.7); 238.9 - 240.9	0.16	**<0.001**
Outer superior	256.1 (16.5); 255.4 - 256.8	253.4 (15.8); 252.6 - 254.3	0.23	**<0.001**
Outer nasal	273.6 (16.5); 272.9 - 274.3	269.8 (16.9); 268.9 - 270.6	0.20	**<0.001**
Outer inferior	254.9 (19.6); 253.9 - 255.3	251.3 (16.7); 250.5 - 252.2	0.20	**<0.001**
Average thickness	266.1 (14.3); 265.5 - 266.7	260.9 (14.1); 260.1 - 261.6	0.31	**<0.001**
Total volume	7.5 (0.40); 7.50 - 7.54	7.37(0.39); 7.35 - 7.39	0.37	**<0.001**

ETDRS: Early Treatment Diabetic Retinopathy Study; SD: standard deviation; CST: central subfield thickness; CI: confidence interval.

Supplementary Table 3. Normative Data for Macular Thickness (in µm) and volume (in mm3) measured by Spectral-Domain Optical Coherence Tomography (Topcon 3SD OCT Master), by DR existence.

	**No DR** (*N*=2849)	**DR** (*N*=561)	**Absolute standardized difference**	**p-value**
**ETDRS macular thickness and volume,** mean (SD); 95% CI				
CST	238.0 (23.4); 237.6-239.9	239.0 (25.4); 236.9-241.2	0.04	0.34
Center	205.1 (31.2); 204.0-206.3	206.6 (33.2); 203.8-209.3	0.04	0.32
Inner temporal	284.9 (16.6); 284.3-285.5	286.0 (17.7); 284.5-287.5	0.06	0.17
Inner superior	296.5 (18.2); 296.9-297.2	296.8 (19.5); 295.1-298.4	0.01	0.77
Inner nasal	299.0 (17.2); 298.4-299.7	298.8 (18.3); 297.2-300.3	0.02	0.72
Inner inferior	292.8 (17.8); 291.1-293.4	293.5 (19.5); 291.9-295.1	0.04	0.4
Outer temporal	243.6 (17.2); 242.9-244.2	246.1 (18.5); 244.6-247.6	0.14	**0.001**
Outer superior	254.5 (16.0); 253.9-255.1	257.2 (17.4); 255.7-258.6	0.16	**<0.001**
Outer nasal	271.8 (16.5); 271.1-272.4	273.1 (18.1); 271.6-274.6	0.08	0.07
Outer inferior	252.9 (16.0); 252.3-253.5	254.6 (17.2); 253.2-256.0	0.10	**0.02**
Average thickness	263.6 (14.1); 263.1-264.1	265.3 (15.2); 264.0-266.5	0.11	**0.01**
Total volume	7.45 (0.40); 7.43-7.56	7.50 (0.42); 7.46-7.53	0.11	**0.01**

ETDRS: Early Treatment Diabetic Retinopathy Study; SD: standard deviation; CST: central subfield thickness; CI: confidence interval; DR: diabetic retinopathy.

Supplementary Table 4. Normative Data for Macular Thickness (in µm) and volume (in mm3) measured by Spectral-Domain Optical Coherence Tomography (Topcon 3SD OCT Master), by type of DM.

	**Type 1 DM** (*N*=275)	**Type 2 DM** (*N*=3135)	**Absolute standardized difference**	**p-value**
**ETDRS macular thickness and volume**, mean (SD); 95% CI				
CST	237.0 (22.9); 242.3-239.7	238.3 (23.8); 237.5-239.1	0.05	0.40
Center	199.3 (27.0); 196.1-202.5	205.9 (30.9); 204.8-207.0	0.22	**<0.001**
Inner temporal	289.5 (16.1); 287.6-291.5	284.7 (16.8); 284.1-285.3	0.29	**<0.001**
Inner superior	303.7 (16.9); 301.7-305.7	295.9 (18.4); 295.3-296.6	**0.44**	**<0.001**
Inner nasal	305.0 (17.4); 303.0-307.1	298.5 (17.3); 297.9-299.1	0.38	**<0.001**
Inner inferior	299.5 (17.9); 297.4-301.7	292.3 (18.0); 291.7-293.5	**0.40**	**<0.001**
Outer temporal	294.7 (16.7); 247.7-251.7	243.5 (17.5); 242.9-244.1	0.36	**<0.001**
Outer superior	263.7 (14.6); 261.9-265.4	254.2 (16.2); 253.6-254.8	**0.61**	**<0.001**
Outer nasal	280.2 (15.6); 278.4-282.1	271.3 (16.7); 270.7-271.8	**0.55**	**<0.001**
Outer inferior	258.6 (14.9); 256.8-260.4	252.7 (16.2); 252.2-253.3	0.38	**<0.001**
Average thickness	270.5 (13.5); 268.8-272.1	263.3 (14.2); 262.8-263.8	**0.51**	**<0.001**
Total volume	7.64 (0.38); 7.6 - 7.7	7.44 (0.40); 7.43 - 7.45	**0.51**	**<0.001**

ETDRS: Early Treatment Diabetic Retinopathy Study; SD: standard deviation; CST: central subfield thickness; CI: confidence interval; DM: Diabetes mellitus.

Supplementary Table 5. Normative Data for Macular Thickness (in µm) and volume (in mm3) measured by Spectral-Domain Optical Coherence Tomography (Topcon 3SD OCT Master), by ~~the~~ years since DM diagnosis.

	**<15 years** (*N*=2553)	**>15 years** (*N*=860)	**Absolute standardized difference**	**p-value**
**ETDRS macular thickness and volume,** mean (SD); 95% CI				
CST	238.6 (23.4); 237.6-239.5	237.1 (24.5); 235.5-238.7	0.06	0.118
Center	205.7 (31.4); 204.5-207.0	204.2 (32.1); 202.1-206.4	0.04	0.24
Inner temporal	285.7 (16.4); 285.1-286.4	283.3 (17.8); 282.1-284.5	0.14	**0.0002**
Inner superior	297.2 (18.3); 296.5-297.6	294.6 (18.6); 293.3-295.8	0.14	**0.0003**
Inner nasal	299.7 (17.2); 299.0-300.4	297.0 (18.0); 295.8-298.2	0.15	**0.0001**
Inner inferior	293.5 (17.6); 292.8-294.2	291.1 (19.2); 289.8-292.4	0.13	**0.0009**
Outer temporal	244.3 (17.3); 243.6-245.0	243.0 (17.9); 241.8-244.2	0.07	0.06
Outer superior	255.4 (16.1); 254.7-256.0	253.8 (16.8); 252.7-254.9	0.09	**0.01**
Outer nasal	272.5 (16.8); 271.8-273.1	270.6 (16.9); 269.5-271.7	0.11	**0.004**
Outer inferior	253.6 (16.1); 252.9-254.2	252.2 (16.6); 251.0-253.3	0.08	**0.02**
Average thickness	264.3 (14.1); 263.8-264.8	262.6 (14.8); 261.6-263.5	0.12	**0.002**
Total volume	7.47 (0.4); 7.45-7.48	7.42 (0.42); 7.39-7.45	0.12	**0.0019**

ETDRS: Early treatment Diabetic retinopathy study; SD: standard deviation; CST: central subfield thickness; CI: confidence interval and DM Diabetes mellitus.
